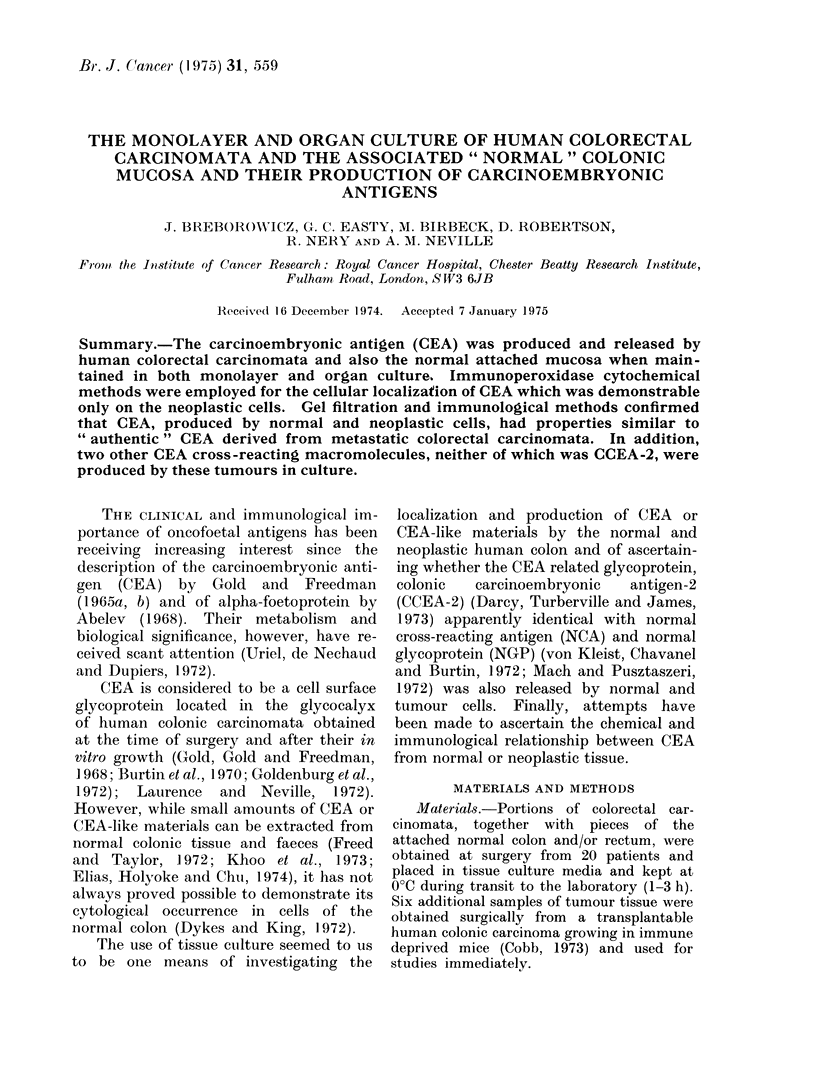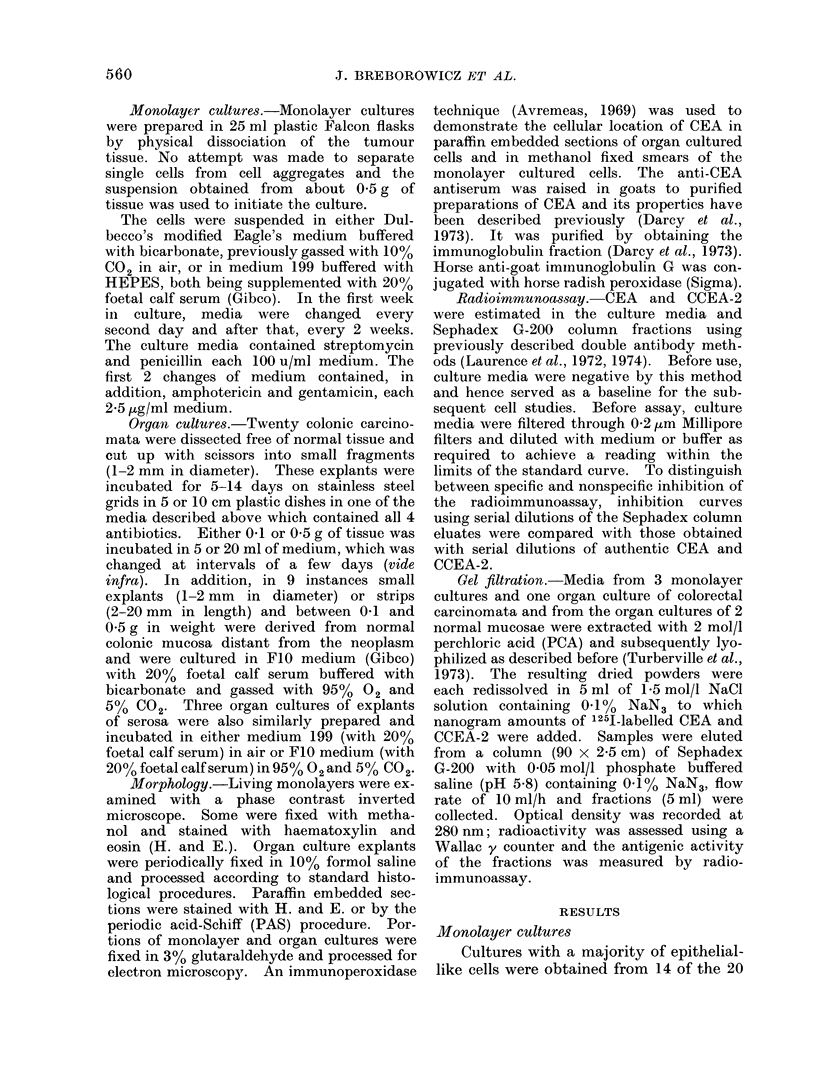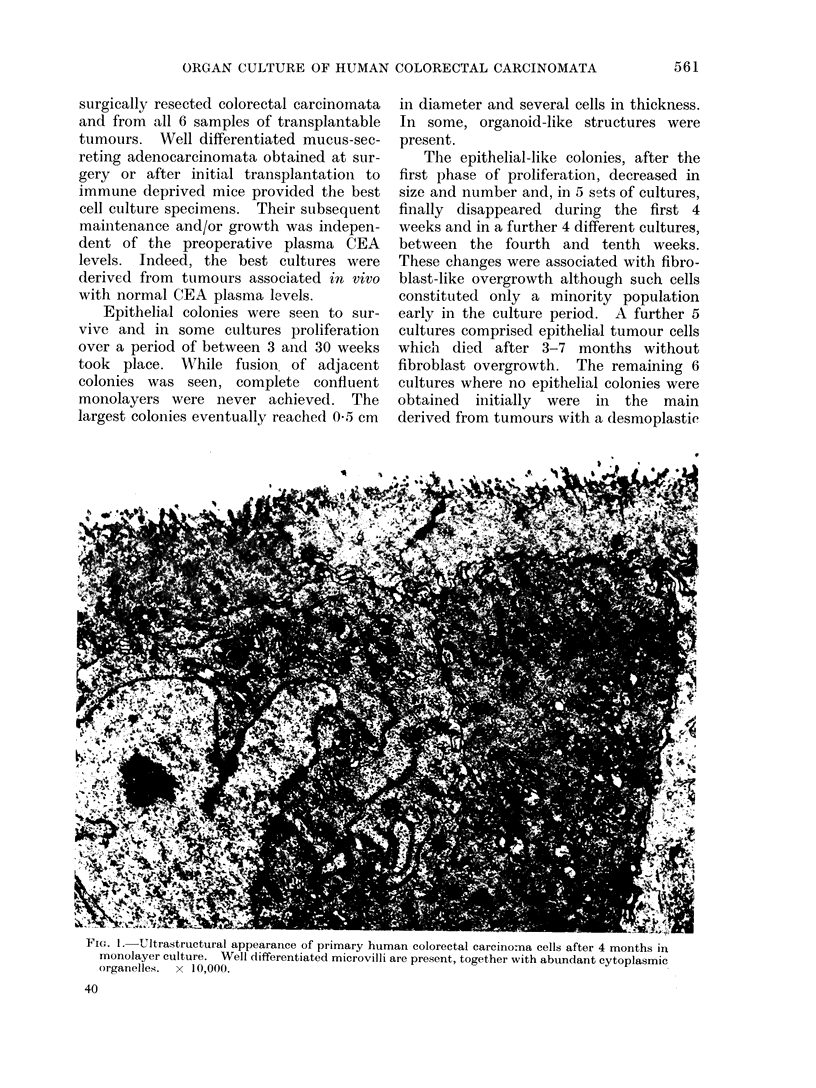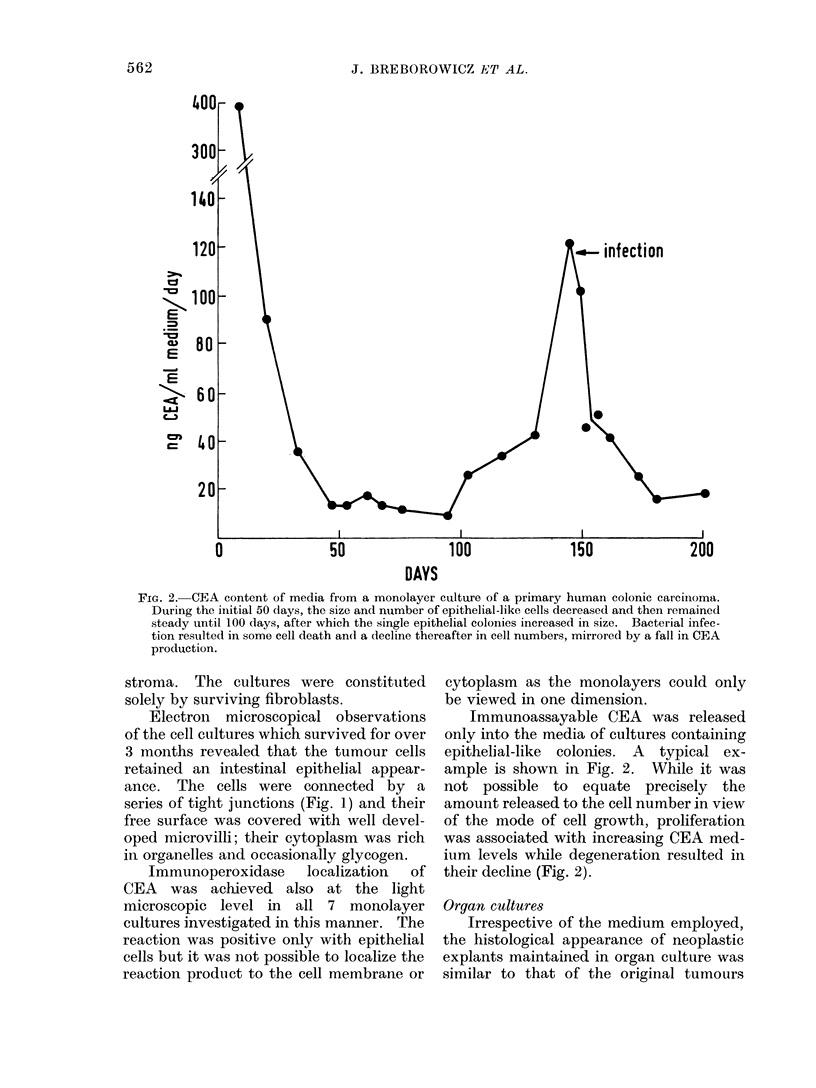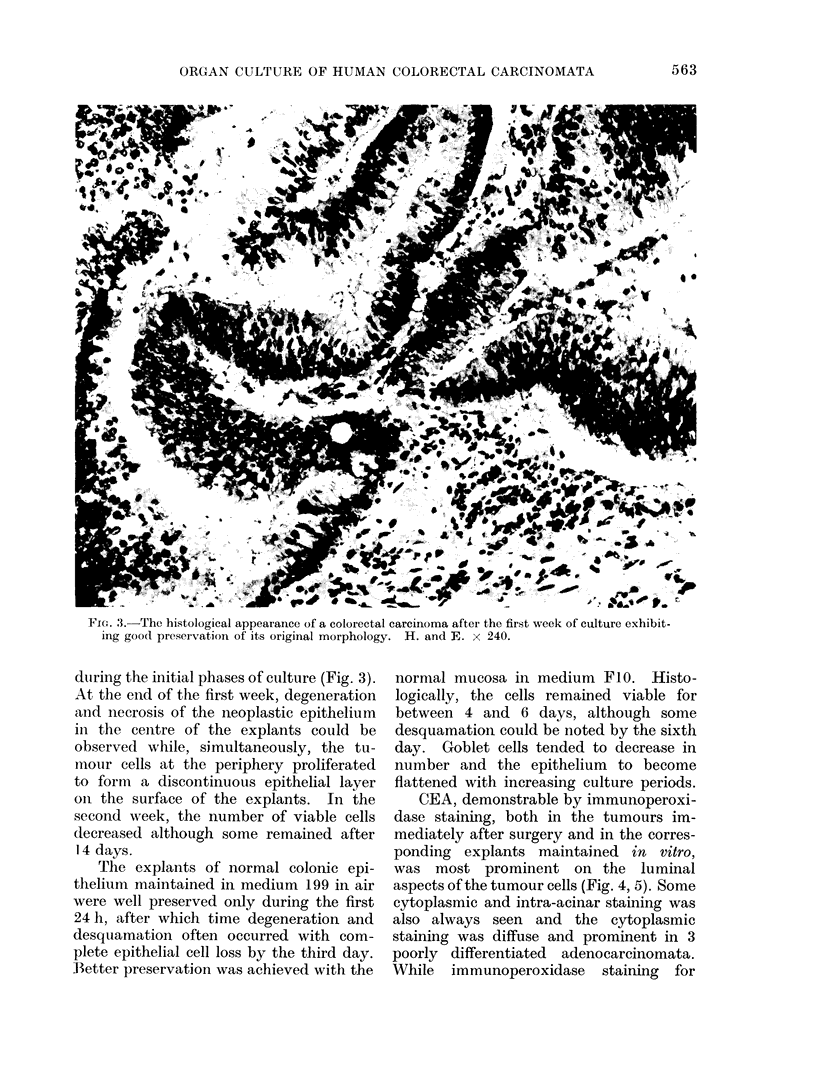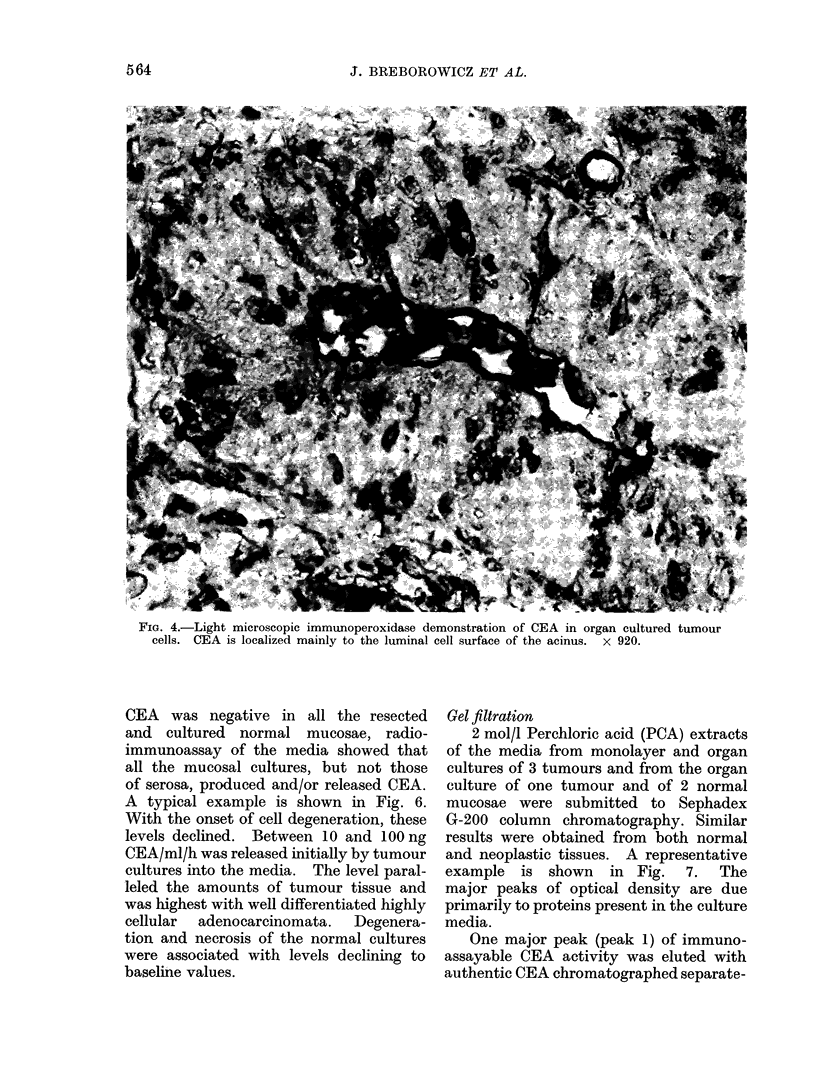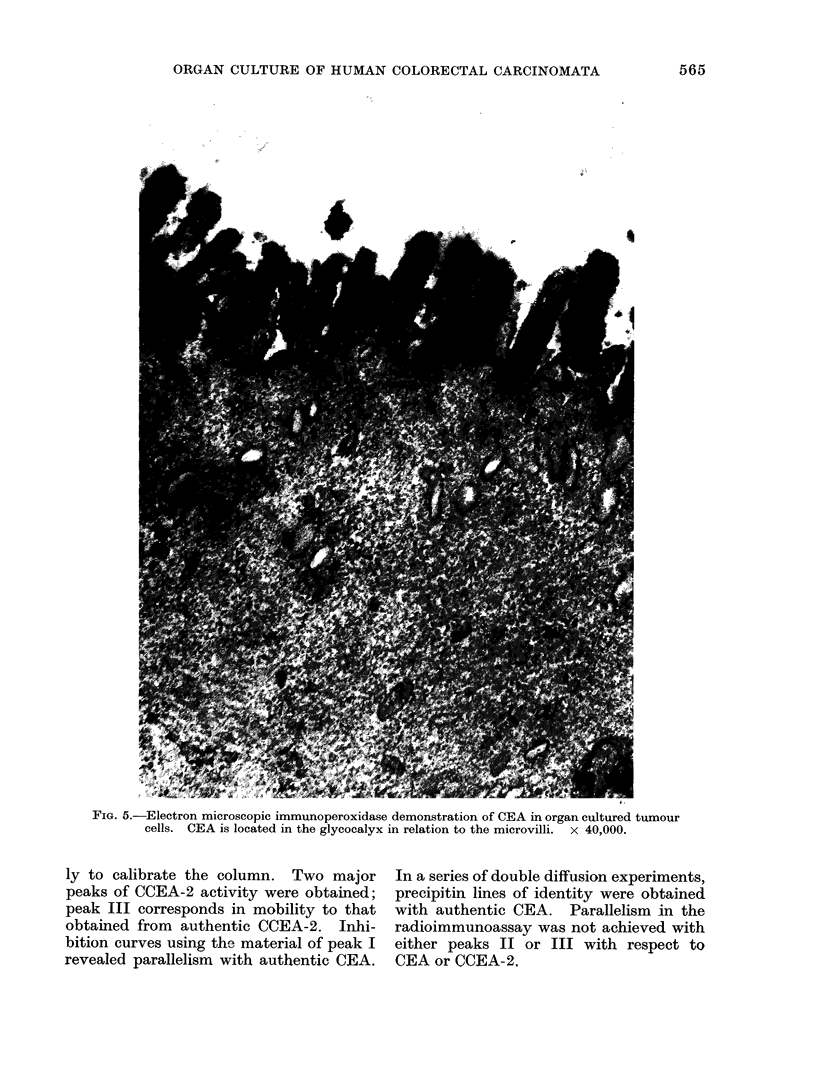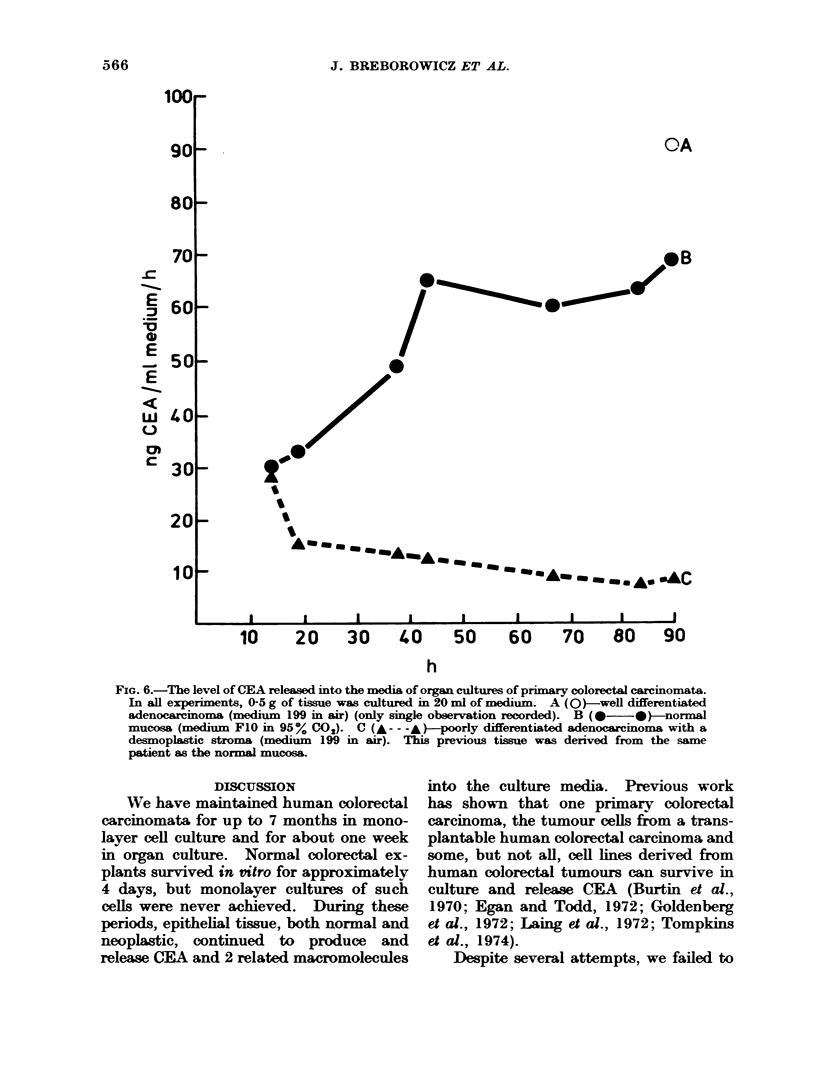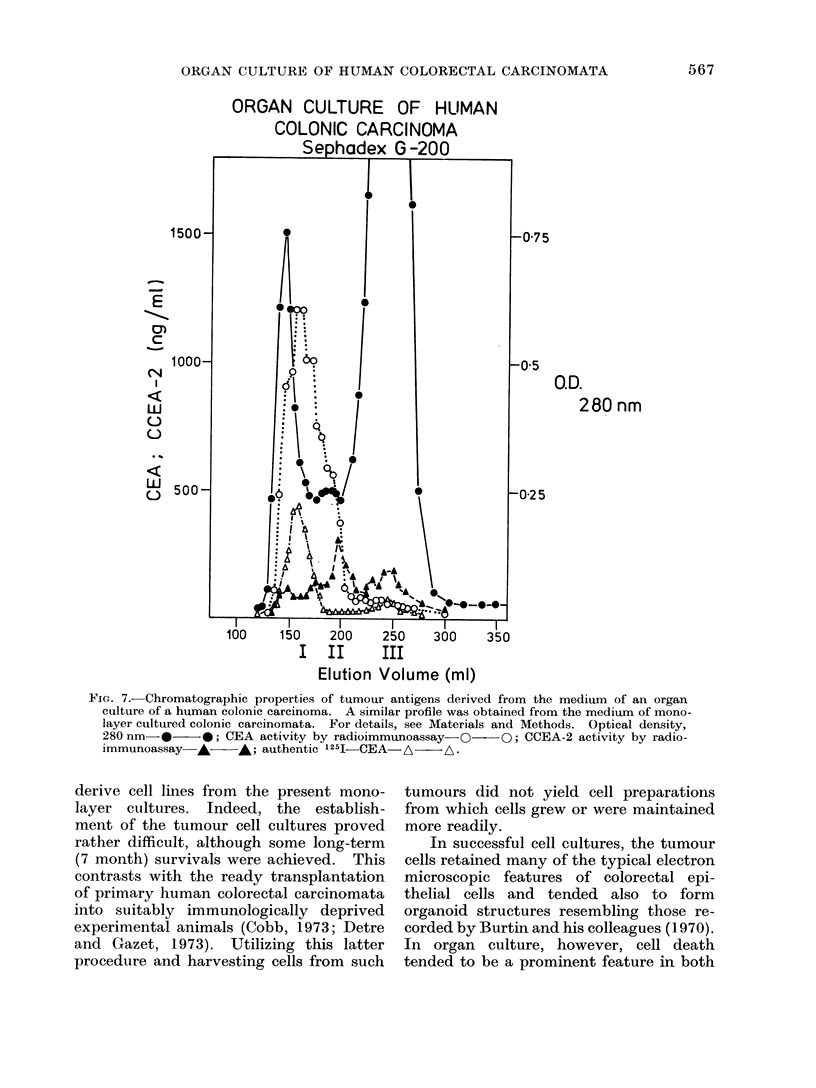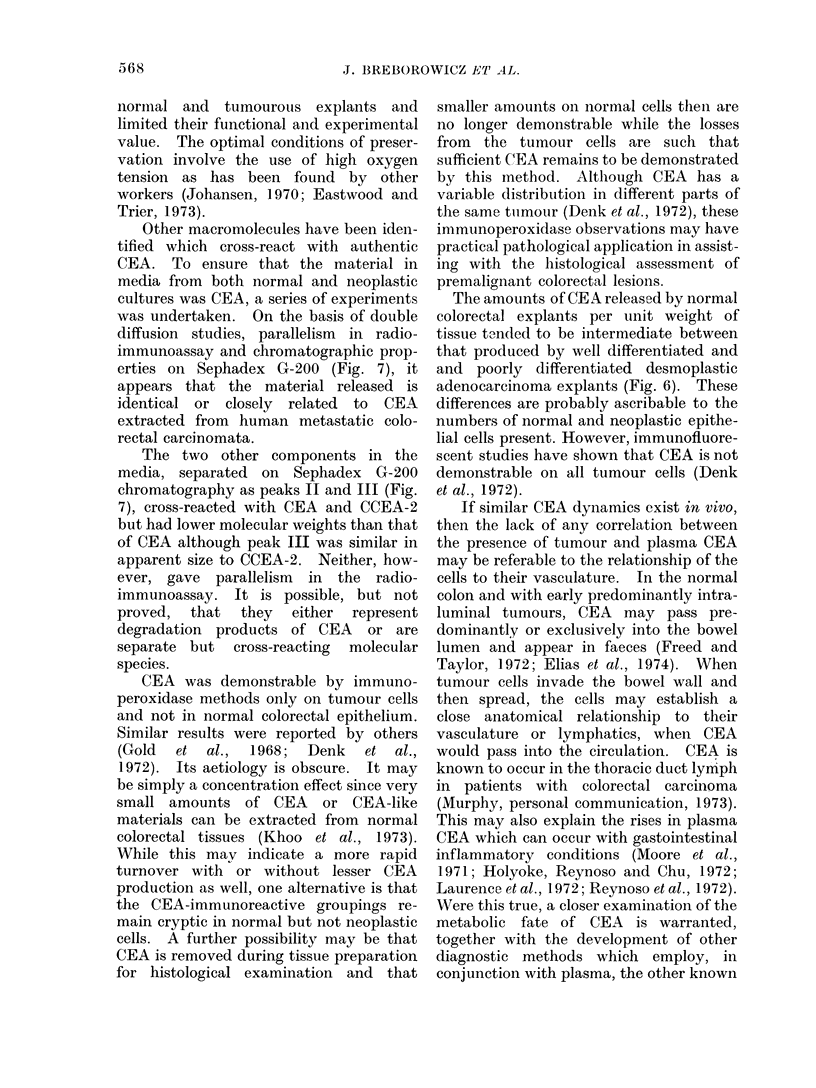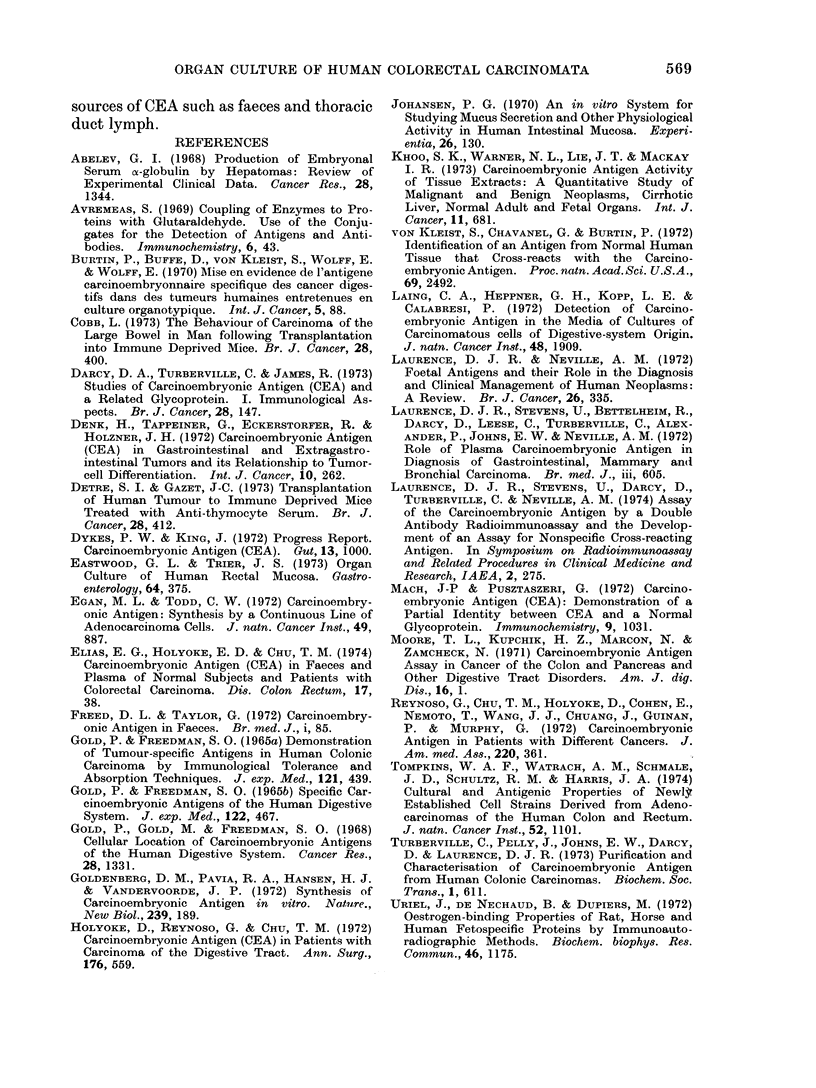# The monolayer and organ culture of human colorectal carcinomata and the associated "normal" colonic mucosa and their production of carcinoembryonic antigens.

**DOI:** 10.1038/bjc.1975.97

**Published:** 1975-05

**Authors:** J. Breborowicz, G. C. Easty, M. Birbeck, D. Robertson, R. Nery, A. M. Neville

## Abstract

**Images:**


*Br. J. Cancer* (1975) **31**, 559

### THE MONOLAYER AND ORGAN CULTURE OF HUMAN COLORECTAL CARCINOMATA AND THE ASSOCIATED " NORMAL " COLONIC MUCOSA AND THEIR PRODUCTION OF CARCINOEMBRYONIC ANTIGENS

J. BREBOROWICZ, G. C. EASTY, M. BIRBECK, D. ROBERTSON, R. NERY AND A. M. NEVILLE

*From the Institute of Cancer Research: Royal Cancer Hospital, Chester Beatty Research Institute, Fulham Road, London, SW3 6JB*

Received 16 December 1974. Accepted 7 January 1975

**Summary.—The carcinoembryonic antigen (CEA) was produced and released by human colorectal carcinomata and also the normal attached mucosa when maintained in both monolayer and organ culture. Immunoperoxidase cytochemical methods were employed for the cellular localization of CEA which was demonstrable only on the neoplastic cells. Gel filtration and immunological methods confirmed that CEA, produced by normal and neoplastic cells, had properties similar to " authentic " CEA derived from metastatic colorectal carcinomata. In addition, two other CEA cross-reacting macromolecules, neither of which was CCEA-2, were produced by these tumours in culture.**

THE CLINICAL and immunological importance of oncofoetal antigens has been receiving increasing interest since the description of the carcinoembryonic antigen (CEA) by Gold and Freedman (1965*a*, *b*) and of alpha-foetoprotein by Abelev (1968). Their metabolism and biological significance, however, have received scant attention (Uriel, de Nechaud and Dupiers, 1972).

CEA is considered to be a cell surface glycoprotein located in the glycocalyx of human colonic carcinomata obtained at the time of surgery and after their *in vitro* growth (Gold, Gold and Freedman, 1968; Burtin *et al.*, 1970; Goldenburg *et al.*, 1972); Laurence and Neville, 1972). However, while small amounts of CEA or CEA-like materials can be extracted from normal colonic tissue and faeces (Freed and Taylor, 1972; Khoo *et al.*, 1973; Elias, Holyoke and Chu, 1974), it has not always proved possible to demonstrate its cytological occurrence in cells of the normal colon (Dykes and King, 1972).

The use of tissue culture seemed to us to be one means of investigating the localization and production of CEA or CEA-like materials by the normal and neoplastic human colon and of ascertaining whether the CEA related glycoprotein, colonic carcinoembryonic antigen-2 (CCEA-2) (Darcy, Turberville and James, 1973) apparently identical with normal cross-reacting antigen (NCA) and normal glycoprotein (NGP) (von Kleist, Chavanel and Burtin, 1972; Mach and Pusztaszeri, 1972) was also released by normal and tumour cells. Finally, attempts have been made to ascertain the chemical and immunological relationship between CEA from normal or neoplastic tissue.

### MATERIALS AND METHODS

*Materials.*—Portions of colorectal carcinomata, together with pieces of the attached normal colon and/or rectum, were obtained at surgery from 20 patients and placed in tissue culture media and kept at 0°C during transit to the laboratory (1–3 h). Six additional samples of tumour tissue were obtained surgically from a transplantable human colonic carcinoma growing in immune deprived mice (Cobb, 1973) and used for studies immediately.

*Monolayer cultures*.—Monolayer cultures were prepared in 25 ml plastic Falcon flasks by physical dissociation of the tumour tissue. No attempt was made to separate single cells from cell aggregates and the suspension obtained from about 0·5 g of tissue was used to initiate the culture.

The cells were suspended in either Dulbecco's modified Eagle's medium buffered with bicarbonate, previously gassed with 10% $CO_2$ in air, or in medium 199 buffered with HEPES, both being supplemented with 20% foetal calf serum (Gibco). In the first week in culture, media were changed every second day and after that, every 2 weeks. The culture media contained streptomycin and penicillin each 100 u/ml medium. The first 2 changes of medium contained, in addition, amphotericin and gentamicin, each 2·5 $\mu$g/ml medium.

*Organ cultures*.—Twenty colonic carcinomata were dissected free of normal tissue and cut up with scissors into small fragments (1–2 mm in diameter). These explants were incubated for 5–14 days on stainless steel grids in 5 or 10 cm plastic dishes in one of the media described above which contained all 4 antibiotics. Either 0·1 or 0·5 g of tissue was incubated in 5 or 20 ml of medium, which was changed at intervals of a few days (*vide infra*). In addition, in 9 instances small explants (1–2 mm in diameter) or strips (2–20 mm in length) and between 0·1 and 0·5 g in weight were derived from normal colonic mucosa distant from the neoplasm and were cultured in F10 medium (Gibco) with 20% foetal calf serum buffered with bicarbonate and gassed with 95% $O_2$ and 5% $CO_2$. Three organ cultures of explants of serosa were also similarly prepared and incubated in either medium 199 (with 20% foetal calf serum) in air or F10 medium (with 20% foetal calf serum) in 95% $O_2$ and 5% $CO_2$.

*Morphology*.—Living monolayers were examined with a phase contrast inverted microscope. Some were fixed with methanol and stained with haematoxylin and eosin (H. and E.). Organ culture explants were periodically fixed in 10% formol saline and processed according to standard histological procedures. Paraffin embedded sections were stained with H. and E. or by the periodic acid-Schiff (PAS) procedure. Portions of monolayer and organ cultures were fixed in 3% glutaraldehyde and processed for electron microscopy. An immunoperoxidase technique (Avremeas, 1969) was used to demonstrate the cellular location of CEA in paraffin embedded sections of organ cultured cells and in methanol fixed smears of the monolayer cultured cells. The anti-CEA antiserum was raised in goats to purified preparations of CEA and its properties have been described previously (Darcy *et al*., 1973). It was purified by obtaining the immunoglobulin fraction (Darcy *et al*., 1973). Horse anti-goat immunoglobulin G was conjugated with horse radish peroxidase (Sigma).

*Radioimmunoassay*.—CEA and CCEA-2 were estimated in the culture media and Sephadex G-200 column fractions using previously described double antibody methods (Laurence *et al*., 1972, 1974). Before use, culture media were negative by this method and hence served as a baseline for the subsequent cell studies. Before assay, culture media were filtered through 0·2 $\mu$m Millipore filters and diluted with medium or buffer as required to achieve a reading within the limits of the standard curve. To distinguish between specific and nonspecific inhibition of the radioimmunoassay, inhibition curves using serial dilutions of the Sephadex column eluates were compared with those obtained with serial dilutions of authentic CEA and CCEA-2.

*Gel filtration*.—Media from 3 monolayer cultures and one organ culture of colorectal carcinomata and from the organ cultures of 2 normal mucosae were extracted with 2 mol/l perchloric acid (PCA) and subsequently lyophilized as described before (Turberville *et al*., 1973). The resulting dried powders were each redissolved in 5 ml of 1·5 mol/l NaCl solution containing 0·1% $NaN_3$ to which nanogram amounts of $^{125}$I-labelled CEA and CCEA-2 were added. Samples were eluted from a column (90 × 2·5 cm) of Sephadex G-200 with 0·05 mol/l phosphate buffered saline (pH 5·8) containing 0·1% $NaN_3$, flow rate of 10 ml/h and fractions (5 ml) were collected. Optical density was recorded at 280 nm; radioactivity was assessed using a Wallac $\gamma$ counter and the antigenic activity of the fractions was measured by radioimmunoassay.

### RESULTS

### *Monolayer cultures*

Cultures with a majority of epithelial-like cells were obtained from 14 of the 20

surgically resected colorectal carcinomata and from all 6 samples of transplantable tumours. Well differentiated mucus-secreting adenocarcinomata obtained at surgery or after initial transplantation to immune deprived mice provided the best cell culture specimens. Their subsequent maintenance and/or growth was independent of the preoperative plasma CEA levels. Indeed, the best cultures were derived from tumours associated *in vivo* with normal CEA plasma levels.

Epithelial colonies were seen to survive and in some cultures proliferation over a period of between 3 and 30 weeks took place. While fusion of adjacent colonies was seen, complete confluent monolayers were never achieved. The largest colonies eventually reached 0·5 cm in diameter and several cells in thickness. In some, organoid-like structures were present.

The epithelial-like colonies, after the first phase of proliferation, decreased in size and number and, in 5 sets of cultures, finally disappeared during the first 4 weeks and in a further 4 different cultures, between the fourth and tenth weeks. These changes were associated with fibroblast-like overgrowth although such cells constituted only a minority population early in the culture period. A further 5 cultures comprised epithelial tumour cells which died after 3–7 months without fibroblast overgrowth. The remaining 6 cultures where no epithelial colonies were obtained initially were in the main derived from tumours with a desmoplastic

FIG. 1.—Ultrastructural appearance of primary human colorectal carcinoma cells after 4 months in monolayer culture. Well differentiated microvilli are present, together with abundant cytoplasmic organelles. × 10,000.

FIG. 2.—CEA content of media from a monolayer culture of a primary human colonic carcinoma. During the initial 50 days, the size and number of epithelial-like cells decreased and then remained steady until 100 days, after which the single epithelial colonies increased in size. Bacterial infection resulted in some cell death and a decline thereafter in cell numbers, mirrored by a fall in CEA production.

stroma. The cultures were constituted solely by surviving fibroblasts.

Electron microscopical observations of the cell cultures which survived for over 3 months revealed that the tumour cells retained an intestinal epithelial appearance. The cells were connected by a series of tight junctions (Fig. 1) and their free surface was covered with well developed microvilli; their cytoplasm was rich in organelles and occasionally glycogen.

Immunoperoxidase localization of CEA was achieved also at the light microscopic level in all 7 monolayer cultures investigated in this manner. The reaction was positive only with epithelial cells but it was not possible to localize the reaction product to the cell membrane or cytoplasm as the monolayers could only be viewed in one dimension.

Immunoassayable CEA was released only into the media of cultures containing epithelial-like colonies. A typical example is shown in Fig. 2. While it was not possible to equate precisely the amount released to the cell number in view of the mode of cell growth, proliferation was associated with increasing CEA medium levels while degeneration resulted in their decline (Fig. 2).

*Organ cultures*

Irrespective of the medium employed, the histological appearance of neoplastic explants maintained in organ culture was similar to that of the original tumours

FIG. 3.—The histological appearance of a colorectal carcinoma after the first week of culture exhibiting good preservation of its original morphology. H. and E. × 240.

during the initial phases of culture (Fig. 3). At the end of the first week, degeneration and necrosis of the neoplastic epithelium in the centre of the explants could be observed while, simultaneously, the tumour cells at the periphery proliferated to form a discontinuous epithelial layer on the surface of the explants. In the second week, the number of viable cells decreased although some remained after 14 days.

The explants of normal colonic epithelium maintained in medium 199 in air were well preserved only during the first 24 h, after which time degeneration and desquamation often occurred with complete epithelial cell loss by the third day. Better preservation was achieved with the normal mucosa in medium F10. Histologically, the cells remained viable for between 4 and 6 days, although some desquamation could be noted by the sixth day. Goblet cells tended to decrease in number and the epithelium to become flattened with increasing culture periods.

CEA, demonstrable by immunoperoxidase staining, both in the tumours immediately after surgery and in the corresponding explants maintained *in vitro*, was most prominent on the luminal aspects of the tumour cells (Fig. 4, 5). Some cytoplasmic and intra-acinar staining was also always seen and the cytoplasmic staining was diffuse and prominent in 3 poorly differentiated adenocarcinomata. While immunoperoxidase staining for

FIG. 4.—Light microscopic immunoperoxidase demonstration of CEA in organ cultured tumour cells. CEA is localized mainly to the luminal cell surface of the acinus. × 920.

CEA was negative in all the resected and cultured normal mucosae, radioimmunoassay of the media showed that all the mucosal cultures, but not those of serosa, produced and/or released CEA. A typical example is shown in Fig. 6. With the onset of cell degeneration, these levels declined. Between 10 and 100 ng CEA/ml/h was released initially by tumour cultures into the media. The level paralleled the amounts of tumour tissue and was highest with well differentiated highly cellular adenocarcinomata. Degeneration and necrosis of the normal cultures were associated with levels declining to baseline values.

*Gel filtration*

2 mol/l Perchloric acid (PCA) extracts of the media from monolayer and organ cultures of 3 tumours and from the organ culture of one tumour and of 2 normal mucosae were submitted to Sephadex G-200 column chromatography. Similar results were obtained from both normal and neoplastic tissues. A representative example is shown in Fig. 7. The major peaks of optical density are due primarily to proteins present in the culture media.

One major peak (peak 1) of immunoassayable CEA activity was eluted with authentic CEA chromatographed separate-

FIG. 5.—Electron microscopic immunoperoxidase demonstration of CEA in organ cultured tumour cells. CEA is located in the glycocalyx in relation to the microvilli. × 40,000.

ly to calibrate the column. Two major peaks of CCEA-2 activity were obtained; peak III corresponds in mobility to that obtained from authentic CCEA-2. Inhibition curves using the material of peak I revealed parallelism with authentic CEA. In a series of double diffusion experiments, precipitin lines of identity were obtained with authentic CEA. Parallelism in the radioimmunoassay was not achieved with either peaks II or III with respect to CEA or CCEA-2.

FIG. 6.—The level of CEA released into the media of organ cultures of primary colorectal carcinomata. In all experiments, 0·5 g of tissue was cultured in 20 ml of medium. A (○)—well differentiated adenocarcinoma (medium 199 in air) (only single observation recorded). B (●——●)—normal mucosa (medium F10 in 95% $CO_2$). C (▲- - -▲)—poorly differentiated adenocarcinoma with a desmoplastic stroma (medium 199 in air). This previous tissue was derived from the same patient as the normal mucosa.

### DISCUSSION

We have maintained human colorectal carcinomata for up to 7 months in monolayer cell culture and for about one week in organ culture. Normal colorectal explants survived *in vitro* for approximately 4 days, but monolayer cultures of such cells were never achieved. During these periods, epithelial tissue, both normal and neoplastic, continued to produce and release CEA and 2 related macromolecules into the culture media. Previous work has shown that one primary colorectal carcinoma, the tumour cells from a transplantable human colorectal carcinoma and some, but not all, cell lines derived from human colorectal tumours can survive in culture and release CEA (Burtin *et al.*, 1970; Egan and Todd, 1972; Goldenberg *et al.*, 1972; Laing *et al.*, 1972; Tompkins *et al.*, 1974).

Despite several attempts, we failed to

FIG. 7.—Chromatographic properties of tumour antigens derived from the medium of an organ culture of a human colonic carcinoma. A similar profile was obtained from the medium of monolayer cultured colonic carcinomata. For details, see Materials and Methods. Optical density, 280 nm—●——●; CEA activity by radioimmunoassay—○——○; CCEA-2 activity by radioimmunoassay—▲——▲; authentic $^{125}$I—CEA—△——△.

derive cell lines from the present monolayer cultures. Indeed, the establishment of the tumour cell cultures proved rather difficult, although some long-term (7 month) survivals were achieved. This contrasts with the ready transplantation of primary human colorectal carcinomata into suitably immunologically deprived experimental animals (Cobb, 1973; Detre and Gazet, 1973). Utilizing this latter procedure and harvesting cells from such tumours did not yield cell preparations from which cells grew or were maintained more readily.

In successful cell cultures, the tumour cells retained many of the typical electron microscopic features of colorectal epithelial cells and tended also to form organoid structures resembling those recorded by Burtin and his colleagues (1970). In organ culture, however, cell death tended to be a prominent feature in both normal and tumourous explants and limited their functional and experimental value. The optimal conditions of preservation involve the use of high oxygen tension as has been found by other workers (Johansen, 1970; Eastwood and Trier, 1973).

Other macromolecules have been identified which cross-react with authentic CEA. To ensure that the material in media from both normal and neoplastic cultures was CEA, a series of experiments was undertaken. On the basis of double diffusion studies, parallelism in radioimmunoassay and chromatographic properties on Sephadex G-200 (Fig. 7), it appears that the material released is identical or closely related to CEA extracted from human metastatic colorectal carcinomata.

The two other components in the media, separated on Sephadex G-200 chromatography as peaks II and III (Fig. 7), cross-reacted with CEA and CCEA-2 but had lower molecular weights than that of CEA although peak III was similar in apparent size to CCEA-2. Neither, however, gave parallelism in the radioimmunoassay. It is possible, but not proved, that they either represent degradation products of CEA or are separate but cross-reacting molecular species.

CEA was demonstrable by immunoperoxidase methods only on tumour cells and not in normal colorectal epithelium. Similar results were reported by others (Gold *et al.*, 1968; Denk *et al.*, 1972). Its aetiology is obscure. It may be simply a concentration effect since very small amounts of CEA or CEA-like materials can be extracted from normal colorectal tissues (Khoo *et al.*, 1973). While this may indicate a more rapid turnover with or without lesser CEA production as well, one alternative is that the CEA-immunoreactive groupings remain cryptic in normal but not neoplastic cells. A further possibility may be that CEA is removed during tissue preparation for histological examination and that smaller amounts on normal cells then are no longer demonstrable while the losses from the tumour cells are such that sufficient CEA remains to be demonstrated by this method. Although CEA has a variable distribution in different parts of the same tumour (Denk *et al.*, 1972), these immunoperoxidase observations may have practical pathological application in assisting with the histological assessment of premalignant colorectal lesions.

The amounts of CEA released by normal colorectal explants per unit weight of tissue tended to be intermediate between that produced by well differentiated and and poorly differentiated desmoplastic adenocarcinoma explants (Fig. 6). These differences are probably ascribable to the numbers of normal and neoplastic epithelial cells present. However, immunofluorescent studies have shown that CEA is not demonstrable on all tumour cells (Denk *et al.*, 1972).

If similar CEA dynamics exist *in vivo*, then the lack of any correlation between the presence of tumour and plasma CEA may be referable to the relationship of the cells to their vasculature. In the normal colon and with early predominantly intraluminal tumours, CEA may pass predominantly or exclusively into the bowel lumen and appear in faeces (Freed and Taylor, 1972; Elias *et al.*, 1974). When tumour cells invade the bowel wall and then spread, the cells may establish a close anatomical relationship to their vasculature or lymphatics, when CEA would pass into the circulation. CEA is known to occur in the thoracic duct lymph in patients with colorectal carcinoma (Murphy, personal communication, 1973). This may also explain the rises in plasma CEA which can occur with gastointestinal inflammatory conditions (Moore *et al.*, 1971; Holyoke, Reynoso and Chu, 1972; Laurence *et al.*, 1972; Reynoso *et al.*, 1972). Were this true, a closer examination of the metabolic fate of CEA is warranted, together with the development of other diagnostic methods which employ, in conjunction with plasma, the other known sources of CEA such as faeces and thoracic duct lymph.

REFERENCES

ABELEV, G. I. (1968) Production of Embryonal Serum α-globulin by Hepatomas: Review of Experimental Clinical Data. *Cancer Res.*, **28**, 1344.

AVREMEAS, S. (1969) Coupling of Enzymes to Proteins with Glutaraldehyde. Use of the Conjugates for the Detection of Antigens and Antibodies. *Immunochemistry*, **6**, 43.

BURTIN, P., BUFFE, D., VON KLEIST, S., WOLFF, E. & WOLFF, E. (1970) Mise en evidence de l'antigene carcinoembryonnaire specifique des cancer digestifs dans des tumeurs humaines entretenues en culture organotypique. *Int. J. Cancer*, **5**, 88.

COBB, L. (1973) The Behaviour of Carcinoma of the Large Bowel in Man following Transplantation into Immune Deprived Mice. *Br. J. Cancer*, **28**, 400.

DARCY, D. A., TURBERVILLE, C. & JAMES, R. (1973) Studies of Carcinoembryonic Antigen (CEA) and a Related Glycoprotein. I. Immunological Aspects. *Br. J. Cancer*, **28**, 147.

DENK, H., TAPPEINER, G., ECKERSTORFER, R. & HOLZNER, J. H. (1972) Carcinoembryonic Antigen (CEA) in Gastrointestinal and Extragastrointestinal Tumors and its Relationship to Tumorcell Differentiation. *Int. J. Cancer*, **10**, 262.

DETRE, S. I. & GAZET, J-C. (1973) Transplantation of Human Tumour to Immune Deprived Mice Treated with Anti-thymocyte Serum. *Br. J. Cancer*, **28**, 412.

DYKES, P. W. & KING, J. (1972) Progress Report. Carcinoembryonic Antigen (CEA). *Gut*, **13**, 1000.

EASTWOOD, G. L. & TRIER, J. S. (1973) Organ Culture of Human Rectal Mucosa. *Gastroenterology*, **64**, 375.

EGAN, M. L. & TODD, C. W. (1972) Carcinoembryonic Antigen: Synthesis by a Continuous Line of Adenocarcinoma Cells. *J. natn. Cancer Inst.*, **49**, 887.

ELIAS, E. G., HOLYOKE, E. D. & CHU, T. M. (1974) Carcinoembryonic Antigen (CEA) in Faeces and Plasma of Normal Subjects and Patients with Colorectal Carcinoma. *Dis. Colon Rectum*, **17**, 38.

FREED, D. L. & TAYLOR, G. (1972) Carcinoembryonic Antigen in Faeces. *Br. med. J.*, i, 85.

GOLD, P. & FREEDMAN, S. O. (1965*a*) Demonstration of Tumour-specific Antigens in Human Colonic Carcinoma by Immunological Tolerance and Absorption Techniques. *J. exp. Med.*, **121**, 439.

GOLD, P. & FREEDMAN, S. O. (1965*b*) Specific Carcinoembryonic Antigens of the Human Digestive System. *J. exp. Med.*, **122**, 467.

GOLD, P., GOLD, M. & FREEDMAN, S. O. (1968) Cellular Location of Carcinoembryonic Antigens of the Human Digestive System. *Cancer Res.*, **28**, 1331.

GOLDENBERG, D. M., PAVIA, R. A., HANSEN, H. J. & VANDERVOORDE, J. P. (1972) Synthesis of Carcinoembryonic Antigen *in vitro*. *Nature., New Biol.*, **239**, 189.

HOLYOKE, D., REYNOSO, G. & CHU, T. M. (1972) Carcinoembryonic Antigen (CEA) in Patients with Carcinoma of the Digestive Tract. *Ann. Surg.*, **176**, 559.

JOHANSEN, P. G. (1970) An *in vitro* System for Studying Mucus Secretion and Other Physiological Activity in Human Intestinal Mucosa. *Experientia*, **26**, 130.

KHOO, S. K., WARNER, N. L., LIE, J. T. & MACKAY I. R. (1973) Carcinoembryonic Antigen Activity of Tissue Extracts: A Quantitative Study of Malignant and Benign Neoplasms, Cirrhotic Liver, Normal Adult and Fetal Organs. *Int. J. Cancer*, **11**, 681.

VON KLEIST, S., CHAVANEL, G. & BURTIN, P. (1972) Identification of an Antigen from Normal Human Tissue that Cross-reacts with the Carcinoembryonic Antigen. *Proc. natn. Acad. Sci. U.S.A.*, **69**, 2492.

LAING, C. A., HEPPNER, G. H., KOPP, L. E. & CALABRESI, P. (1972) Detection of Carcinoembryonic Antigen in the Media of Cultures of Carcinomatous cells of Digestive-system Origin. *J. natn. Cancer Inst.*, **48**, 1909.

LAURENCE, D. J. R. & NEVILLE, A. M. (1972) Foetal Antigens and their Role in the Diagnosis and Clinical Management of Human Neoplasms: A Review. *Br. J. Cancer*, **26**, 335.

LAURENCE, D. J. R., STEVENS, U., BETTELHEIM, R., DARCY, D., LEESE, C., TURBERVILLE, C., ALEXANDER, P., JOHNS, E. W. & NEVILLE, A. M. (1972) Role of Plasma Carcinoembryonic Antigen in Diagnosis of Gastrointestinal, Mammary and Bronchial Carcinoma. *Br. med. J.*, iii, 605.

LAURENCE, D. J. R., STEVENS, U., DARCY, D., TURBERVILLE, C. & NEVILLE, A. M. (1974) Assay of the Carcinoembryonic Antigen by a Double Antibody Radioimmunoassay and the Development of an Assay for Nonspecific Cross-reacting Antigen. In *Symposium on Radioimmunoassay and Related Procedures in Clinical Medicine and Research, IAEA*, **2**, 275.

MACH, J-P & PUSZTASZERI, G. (1972) Carcinoembryonic Antigen (CEA): Demonstration of a Partial Identity between CEA and a Normal Glycoprotein. *Immunochemistry*, **9**, 1031.

MOORE, T. L., KUPCHIK, H. Z., MARCON, N. & ZAMCHECK, N. (1971) Carcinoembryonic Antigen Assay in Cancer of the Colon and Pancreas and Other Digestive Tract Disorders. *Am. J. dig. Dis.*, **16**, 1.

REYNOSO, G., CHU, T. M., HOLYOKE, D., COHEN, E., NEMOTO, T., WANG, J. J., CHUANG, J., GUINAN, P. & MURPHY, G. (1972) Carcinoembryonic Antigen in Patients with Different Cancers. *J. Am. med. Ass.*, **220**, 361.

TOMPKINS, W. A. F., WATRACH, A. M., SCHMALE, J. D., SCHULTZ, R. M. & HARRIS, J. A. (1974) Cultural and Antigenic Properties of Newly Established Cell Strains Derived from Adenocarcinomas of the Human Colon and Rectum. *J. natn. Cancer Inst.*, **52**, 1101.

TURBERVILLE, C., PELLY, J., JOHNS, E. W., DARCY, D. & LAURENCE, D. J. R. (1973) Purification and Characterisation of Carcinoembryonic Antigen from Human Colonic Carcinomas. *Biochem. Soc. Trans.*, **1**, 611.

URIEL, J., DE NECHAUD, B. & DUPIERS, M. (1972) Oestrogen-binding Properties of Rat, Horse and Human Fetospecific Proteins by Immunoautoradiographic Methods. *Biochem. biophys. Res. Commun.*, **46**, 1175.